# Peripheral IgE Repertoires of Healthy Donors Carry Moderate Mutation Loads and Do Not Overlap With Other Isotypes

**DOI:** 10.3389/fimmu.2019.01543

**Published:** 2019-07-03

**Authors:** Marvyn T. Koning, Ignis J. M. Trollmann, Cornelis A. M. van Bergen, Diego Alvarez Saravia, Marcelo A. Navarrete, Szymon M. Kiełbasa, Hendrik Veelken

**Affiliations:** ^1^Department of Hematology, Leiden University Medical Center, Leiden, Netherlands; ^2^School of Medicine, University of Magallanes, Punta Arenas, Chile; ^3^Department of Biomedical Data Sciences, Leiden University Medical Center, Leiden, Netherlands

**Keywords:** affinity maturation, allergy, B-cell receptor, class switching, IgE

## Abstract

IgE-mediated allergic disease represents an increasing health problem. Although numerous studies have investigated IgE sequences in allergic patients, little information is available on the healthy IgE repertoire. IgM, IgG, IgA, and IgE transcripts from peripheral blood B cells of five healthy, non-atopic individuals were amplified by unbiased, template-switching, isotype-specific PCR. Complete VDJ regions were sequenced to near-exhaustion on the PacBio platform. Sequences were analyzed for clonal relationships, degree of somatic hypermutation, IGHV gene usage, evidence of antigenic selection, and N-linked glycosylation motifs. IgE repertoires appeared to be highly oligoclonal with preferential usage of certain IGHV genes compared to the other isotypes. IgE sequences carried more somatic mutations than IgM, yet fewer than IgG and IgA. Many IgE sequences contained N-linked glycosylation motifs. IgE sequences had no clonal relationship with the other isotypes. The IgE repertoire in healthy individuals is derived from relatively few clonal expansions without apparent relations to immune reactions that give rise to IgG or IgA. The mutational burden of normal IgE suggests an origin through direct class-switching from the IgM repertoire with little evidence of antigenic drive, and hence presumably low affinity for specific antigens. These findings are compatible with a primary function of the healthy IgE repertoire to occupy Fcε receptors for competitive protection against mast cell degranulation induced by allergen-specific, high-affinity IgE. This background knowledge may help to elucidate pathogenic mechanisms in allergic disease and to design improved desensitization strategies.

## Introduction

Type I hypersensitivity is an immune response triggered by allergen-specific IgE. Binding of allergen-bound IgE to Fcε receptors on mast cells leads to prompt degranulation, which provokes a range of clinical symptoms including atopic dermatitis, asthma, allergic rhinoconjunctivitis, urticarial, and anaphylaxis (1, 2). Despite the recognition that IgE-mediated diseases are becoming an ever increasing health burden, especially in urban societies (3), the mechanisms leading to IgE-mediated disease, as well as the underlying principles causing clinical heterogeneity, remain incompletely understood (4, 5). An improved understanding of the development of IgE repertoires in healthy individuals may aid in the identification of these disease mechanisms and facilitates efficient design of anti-allergic strategies (6, 7).

Two distinct pathways to generate IgE have been identified in mouse models. In the direct pathway, functional B-cell receptor (BCR) genes undergo direct class-switch recombination (CSR) from IgM to IgE isotype and yield predominantly IgE with low affinity to antigen. In the indirect pathway, high-affinity IgE results from secondary CSR in B cells that express IgG or potentially IgA (8, 9). Along with increased affinity, IgE derived from IgG1 B cells also carry significantly more mutations than IgE in IgG1-deficient mice (10). The existence of multiple pathways has been further supported by flow cytometric analyses in humans showing subsets both dependent and independent of germinal centers (11).

Since low and high affinity IgE compete for occupation of the Fcε receptor, higher concentrations of low-affinity IgE than high-affinity IgE can theoretically provide protection against anaphylaxis (10). In non-allergic humans, very limited information on the extent and origin of the IgE repertoire is currently available and consists of only 60 near-full-length IgE sequences from two individuals (12). Although recently a number of studies have applied massive parallel sequencing to gain new insights in the IgE repertoire, none of them generated the full-length sequences essential for comprehensive analysis and/or used primer binding bias-free methodology (13–18).

This study aimed to provide in-depth characterization of the IgE repertoire in healthy, non-allergic individuals as an essential reference for comparative studies in allergic and desensitized individuals. We determined the peripheral blood IgE BCR repertoire of five non-allergic donors to near completion by unbiased, full-length massive parallel sequencing. IgE BCR repertoires were compared to IgM, IgG, and IgA repertoires to support the hypothesis that the direct pathway of IgE generation would be dominant in non-allergic human individuals.

## Materials and Methods

### Material Collection and Storage

Cryopreserved aliquots of Ficoll-separated mononuclear cells (PBMC) from five healthy, asymptomatic stem cell donors were obtained from the biobank of the Leiden University Medical Center biobank in accordance with local guidelines. Absence of atopic constitution was confirmed by measurement of total IgM, IgG, IgA, and IgE immunoglobulin levels in time-matched serum samples.

### Flow Cytometry Analysis for Expression of CD19 and IgE

IgE-expressing B cells were isolated from aliquots of 1 × 10^5^ thawed PBMC by flow cytometry. To avoid artifacts from binding of IgE to Fcε receptor-expressing cells (19), cells were fixed in 200 μl 1% paraformaldehyde in phosphate-buffered saline (B. Braun, Melsungen, Germany) with 5% fetal bovine serum (Bodinco, Alkmaar, The Netherlands) and 0.2% saponin (Sigma Aldrich, St. Louis, MO, USA) for 8 min at 4°C. After washing, fixed cells were permeabilized for 30 min at 4°C in 200 μl of the same buffer without paraformaldehyde. Cells were washed once more and stained with anti-CD19-FITC (BD Biosciences, Franklin Lakes, NJ, USA) and anti-IgE-APC (Miltenyi Biotec, Leiden, The Netherlands) for 30 min at 4°C in the dark, followed by another wash and resuspension. The abundance of CD19^+^ and IgE^+^ cells was determined by flow cytometry in live gated cells.

### BCR Repertoire Sequencing

The ARTISAN PCR protocol for unbiased amplification of BCR repertoires (20) was adapted for IgE by designing a series of IgE constant region-specific reverse primers (ε.rt 5′-GGCATAGTGACCAGAGAGCG-3′ for reverse transcription; ε.pcr1 5′-GGTCACCATCACCGGCTCCG-3′ for initial PCR amplification; ε.pcr2 5′-GGCAGCCCAGAGTCACGG-3′ for semi-nested PCR amplification; ε.bc 5′-[barcode]-CGGATGGGCTCTGTGTGG-3′ for barcoding; barcodes: 5′-CCATCTCATATGTAGTACTCT-3′, 5′-CGGATGGGCTCTGTGTGG-3′, 5′-CGGATGGGCTCTGTGTGG-3′, 5′-CGGATGGGCTCTGTGTGG-3′ and 5′-CGGATGGGCTCTGTGTGG-3′). B cells were isolated from aliquots of thawed cells by removal of non-B cells with magnetic beads (B cell isolation kit II; Miltenyi Biotec, Leiden, The Netherlands) and routinely yielded a purity of >99% CD19^+^ B cells as assessed by flow cytometry. For each healthy donor, 2 × 10^6^ B cells were divided into five aliquots.

Messenger RNA isolation and cDNA synthesis were performed separately for each aliquot with addition of the ε.rt primer to the ARTISAN cDNA synthesis mix (20). IgM, IgG, and IgA cDNA was amplified according to the original ARTISAN PCR protocol. Due to the low abundance of IgE^+^ B cells in healthy donor peripheral blood, amplification of functional IgE transcripts was extensively optimized with different primer combinations on serial dilutions (100–0.01%) of the IgE-expressing multiple myeloma cell line U266 (DSMZ, Braunschweig, Germany) in a background of healthy donor PBMCs. For IgE repertoires, cDNA was first amplified for 15 cycles with the ε.pcr1 primer. First-round IgE amplicons were purified by silica spin columns (Promega, Madison, WI, USA) and re-amplified for 15 cycles of semi-nested PCR using the ε.pcr2 primer. Libraries were barcoded at the 5′ terminus to identify the donor and at the 3′ terminus to identify individual aliquots. Pooled libraries were amplified as single molecules in rolling circles on a total of five SMRT cells on the RSII system (Pacific Biosciences, Menlo Park, CA, USA). IgE sequences were sequenced to high depth to achieve near-complete representation of all IgE^+^ B cells present in the sample.

### Sequence and Statistical Analysis

Output sequence files were filtered with SMRT portal software for a minimum of eight sequencing passes. All sequences were annotated by IMGT HighV-QUEST (21). For statistical analyses, sequences with identical IGHV genes and amino acid CDR3 sequences within one aliquot were counted as a single sequence.

IGHV gene usage was compared between isotypes by Fisher's exact test and corrected for multiple testing by Bonferroni correction. Cumulative differences in IGHV gene usage were calculated by determining the absolute differences in fractional IGHV usage between two isotypes for every IGHV gene and adding these to a cumulative difference with a theoretical maximum of 200. BCR mutational status and CDR3 length were compared between isotypes by unpaired *t*-test.

N-linked glycosylation motifs were identified as Asn-X-Ser/Thr motifs (where X may be any amino acid except proline) and their abundance, location, and the mutational status of the corresponding sequence were compared between isotypes using unpaired t and Fisher's exact tests.

Clonal B-cell expansions were defined as the presence of BCR sequences with identical V, D and J gene usage, identical CDR3 length and ≥95% nucleotide overlap in CDR3 in either multiple aliquots or in more than one isotype of any individual donor. Intraclonal sequence variation was defined as the distance from the clonal consensus and determined for the largest 10 IgE clones, as well as the largest 5 IgM, IgG, and IgA clones.

### Calculation of Sampling Depth

To assess how many cells from the sample were represented in the VDJ sequence libraries, we performed an *in silico* simulation to estimate the fraction of observed unique sequences sampled out of a large pool of sequences. A unique numerical identifier was assigned to each unique observed BCR sequence for each donor, and 5 × 10^5^ copies of every identifier were pooled in the simulation. Consequently, random sequences were sampled from the pool up to the number of sequences obtained from massive parallel sequencing, and the number of unique identifiers was counted. This simulation was performed 100 times per donor, and the median number of unique samples identifiers per donor was calculated.

## Results

According to applicable stem cell donor regulations, all donors had no atopic constitution, denied any allergic symptoms, and had normal serum immunoglobulin and IgE concentrations (Table 1).

**Table 1:** Serum immunoglobulin concentrations in five healthy donors.

**Subject**	**IgM (g/L)**	**IgG (g/L)**	**IgA (g/L)**	**IgE (kU/L)**
1	1.56	8.67	2.17	12.4
2	1.19	9.68	1.93	76.8
3	0.38	8.02	1.32	41.8
4	0.53	9.20	1.78	23.7
5	0.67	6.45	0.61	14.9

### Sequence Acquisition

A median of 0.09% of peripheral blood B cells of healthy donors expressed IgE (Table 2), corresponding to a median of 2,000 cells per 2 × 10^6^ B cells. A median of 3,254 total full-length, potentially functional VDJ IgE sequences were obtained per donor by ARTISAN PCR and deep PacBio sequencing, representing a median of 1.52 IgE sequences per IgE^+^ B cell present in the sample. *In silico* simulations indicated that this amount of oversampling would theoretically return a median of 78% of unique sequences (Supplementary Data). In addition, medians of 2,088 IgM (range: 1,287–2,437), 1,221 IgG (range: 735–2,144), and 2,770 IgA (range: 1,351–3,012) unique, full-length and potentially functional VDJ sequences were obtained per donor.

**Table 2:** Quantification of IgE^+^ B cells and sequencing depth.

	**% CD19^**+**^**	**IgE**^****+****^ **B cells**		**VDJ sequences**		**Median theoretical**
**Donor**	**cells within live gate**	**% within CD19^**+**^ gate**	**per 2 × 10^**6**^ CD19^**+**^ cells**	**Total**	**per cell**	**coverage (range)**
1	11.3	0.10	2,000	3,254	1.63	81% (79–82)
2	10.8	0.09	1,800	1,814	1.01	63% (62–66)
3	10.8	0.15	3,000	3,716	1.24	71% (69–73)
4	9.2	0.06	1,200	1,823	1.52	78% (76–81)
5	6.5	0.08	1,600	5,199	3.25	96% (95–97)
Median	10.8	0.09	2,000	3,254	1.52	78%

*Coverage represents the amount of unique sequences sampled from the pool in an in silico simulation assuming no PCR amplification bias. Its range is based on the minimum and maximum values obtained from 100 simulations*.

### Clonal B-Cell Expansions

To identify and quantify clonal B cell expansions, we first assigned all individual VDJ sequences within an isotype of each donor to clonotypes according to stringent criteria developed rationally to minimize the calling of false-positive clonal relationships (Supplementary Data). This analysis identified a median of 39 (range: 23–46) putative unique IgE clonotypes per donor and indicated representation of each clonotype by an average of 85 closely related VDJ sequences. Additional manual inspection revealed that some of these putative clonotypes shared substantial numbers of individual mutations in their IGHV, strongly suggesting a common clonal origin despite less than 95% CDR3 identity and despite occasional variation in CD3 length. Therefore, we combined putative clonotypes from an individual that shared at least 70% of their IGHV mutations regardless of CDR3 similarities into definitive clonotypes for all further analyses. We identified a total of 146 definitive IgE clonotypes (median per donor 31; range: 18–38). In a single instance, these criteria indicated the presence of a single clonotype in two donors. Since the libraries of these two donors were sequenced on the same SMRT cell, this phenomenon may originate from barcode contamination and does not permit to conclude the presence of canonical IgE clonotypes across individuals.

**Figure 1:**
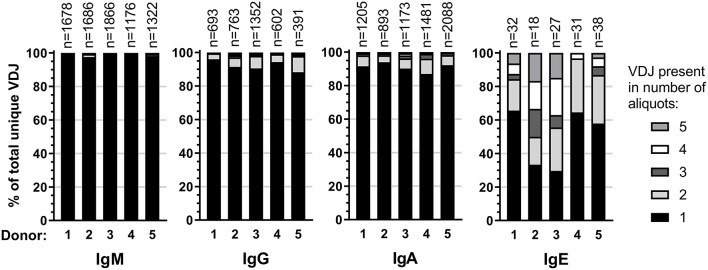
Clonality in the healthy BCR repertoire. Distribution of unique VDJ sequences present in one or more cellular aliquots per isotype and donor.

**Figure 2:**
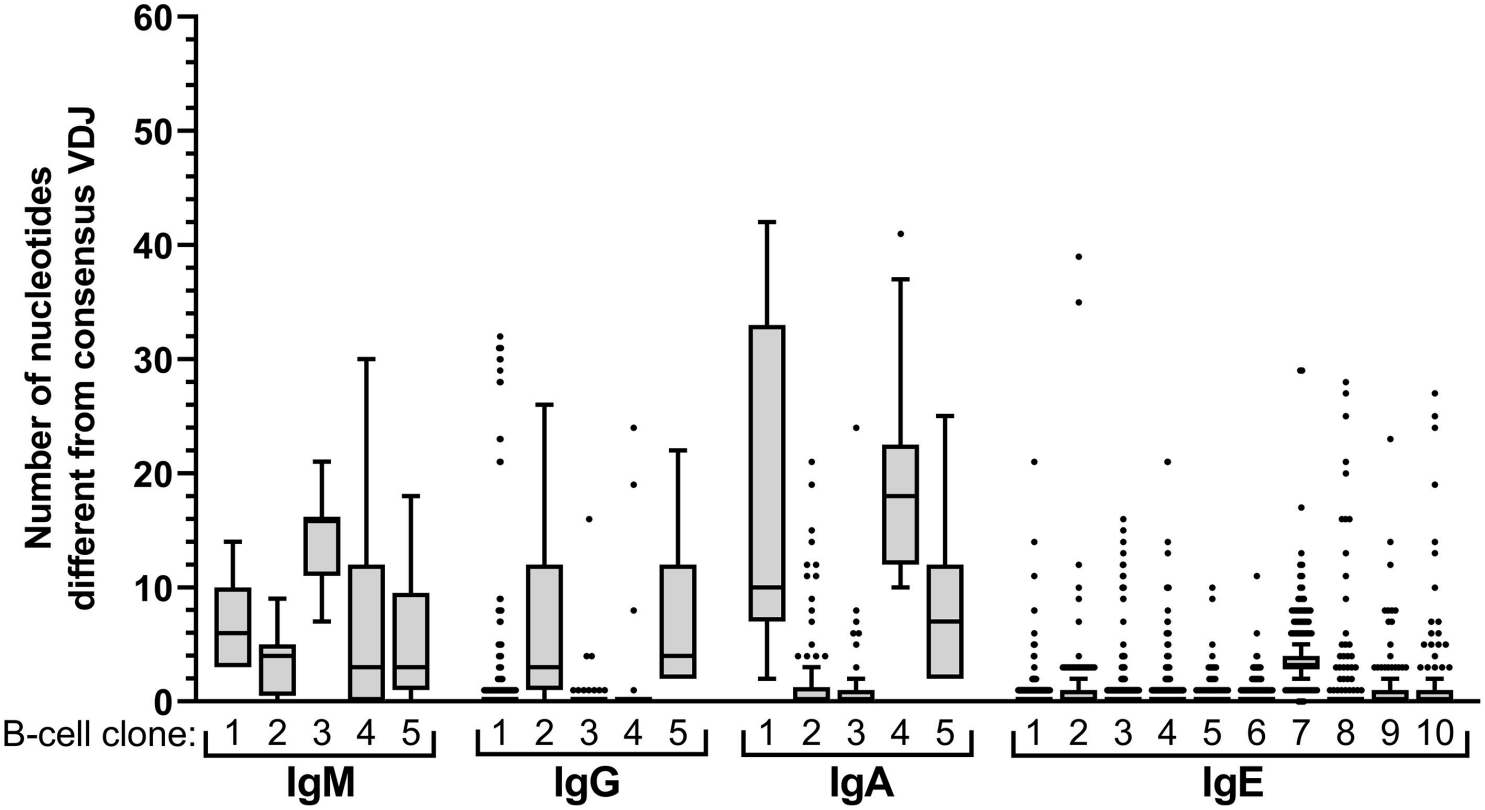
Intraclonal sequence diversity in the healthy BCR repertoire. Intraclonal variability per isotype. The number of nucleotide differences compared to the respective consensus VDJ sequence is displayed for every VDJ sequence of the 5 largest B-cell clones expressing IgM, IgG, or IgA, respectively, and for the 10 largest IgE-expressing B-cell clones.

Expansion of an IgE-expressing B-cell clone was unequivocally demonstrated by the presence of 69 of the total of 146 IgE clonotypes (47%) in at least two B-cell aliquots (Figure 1), corresponding to a median of 12 (range: 11–19) clonal expansions of IgE-expressing B-cells per donor. In comparison, only 1.3 of IgM, 7.9 of IgG, and 9.1% of IgA VDJ clonotypes were found in multiple aliquots and therefore derived from clonally expanded B cells, corresponding to a median of 14 (range: 6–40; total 97), 46 (range: 28–128; total 301) and 115 (range: 54–192; total 625) clonal expansions per donor and isotype, respectively (Figure 1). IgE clonal expansions were fewer than IgG (*p* = 0.034) and IgA (*p* = 0.0017), but similar to IgM expansions (*p* = 0.39). Intraclonal sequence diversity was lower in IgE than in all other isotypes (IgM: *p* = 0.0002; IgG: *p* = 0.0184; IgA: *p* = 0.0039; Figure 2). Twelve VDJ clonotypes were present in both IgM and IgG BCR repertoires, 24 in IgM and IgA, and 45 in IgG and IgA. Seven clonotypes comprised IgM, IgG, and IgA isotypes. In marked contrast, no relationship was found between any IgE and non-IgE VDJ. Since this finding is in contrast with previous reports (22), we performed extensive *in silico* simulations that demonstrated frequent false-positive conclusions of clonal overlap when inappropriately relaxed criteria for clonal relationship are applied (Supplementary Data).

### BCR Characteristics per Isotype

**Figure 3:**
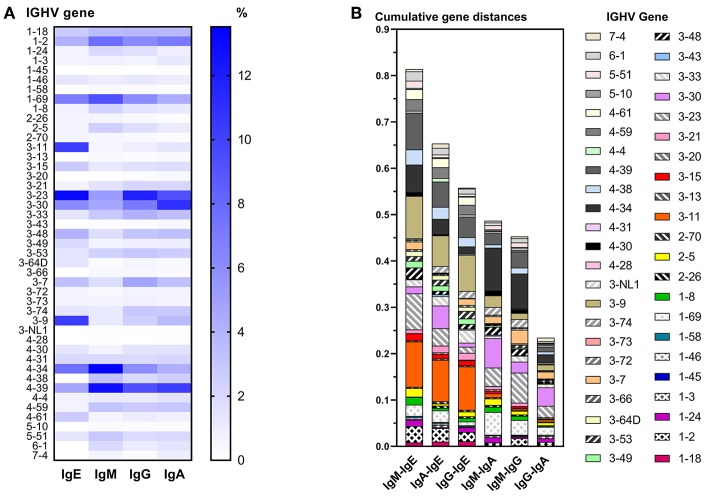
Differential IGHV usage between isotypes. **(A)** Heat map indicating the relative frequency of all IGHV genes among unique VDJ sequences per isotype. For each isotype, the frequencies shown add up to 100%. **(B)** Pairwise comparison of cumulative IGHV gene distance between two isotypes. For each IGHV gene, the fractions of VDJ sequences containing that IGHV gene within an isotype were calculated, and the numerical difference of these fractions between two isotypes was determined per IGHV gene. For each pairwise comparison between isotypes, all differences of IGHV gene fractions were added to obtain their cumulative distance.

IgE VDJ utilized more frequently IGHV3-9 and IGHV3-11 (*p* < 0.0001), and less frequently IGHV4-39 than the remaining isotypes (*p* = 0.0018; Figure 3A). Concordantly, cumulative gene fraction distances between IgE and other isotypes were significantly larger than distances between IgM and IgG, IgM, and IgA, and IgG, and IgA (Figure 3B).

**Figure 4:**
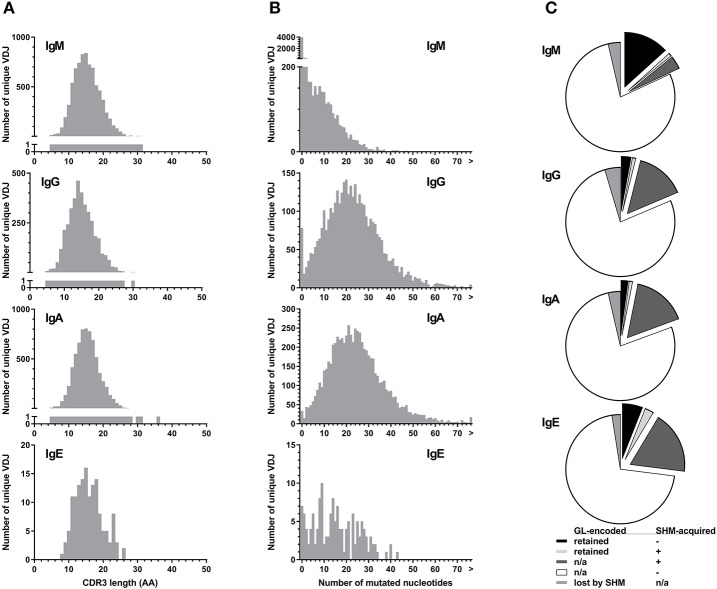
Parameters of BCR hypervariability. **(A)** Comparison of CDR3 length between isotypes. The number of unique VDJ sequences with a given CDR3 amino acid (AA) length as defined by IGMT HighV-Quest is shown per isotype. **(B)** Comparison of somatic hypermutation between isotypes. The number of unique VDJ sequences with a given number of nucleotide changes compared to their most closely related germ-line IGHV sequence is displayed per isotype. Sequences longer than 75 bp are indicated by “>”. **(C)** Fractions of VDJ sequences with N-glycosylation motifs per isotype. Fractions indicate the origin of the N-glycosylation motifs and whether a germ-line (GL)-encoded motif was lost by somatic hypermutation (SHM).

All isotypes had median CDR3 lengths of 15 amino acids (Figure 4A). Unique IgE VDJ carried fewer mutations (4.9%) than the other class-switched isotypes (*p* < 0.001) (Figure 4B).

Despite their moderate SHM burden, more IgE VDJ (23%) had acquired N-linked glycosylation motifs (NLGM) through SHM than IgG (16%; *p* < 0.001) and IgA (17%; *p* < 0.001). When considering all NLGM, i.e., both germline-encoded (from IGHV1-8, IGHV4-34, and IGHV5-10-1) and SHM-derived motifs, their prevalence of 27% in all IgE VDJ was significantly higher (*p* < 0.0001) than in IgM, IgG, and IgA (Figure 4C). This difference was largely attributable to preservation of germ-line-encoded NLGM in the IgE compartment (Table 3).

**Table 3:** Preservation of germline-encoded N-linked glycosylation sites.

	**Retention of germline N-linked glycosylation motifs**		
**Isotype**	**IGHV1-8**	**IGHV4-34**	**IGHV5-10-1**
IgM	87%	89%	83%
IgG	51%	47%	43%
IgA	59%	44%	62%
IgE	94%	64%	N/A

*No IGHV5-10-1-containing VDJ were found for IgE*.

## Discussion

In conclusion, we provide the first comprehensive inventory of near-complete peripheral blood IgE repertoires from healthy individuals through tailored methodology that lacks primer binding bias, yields full-length VDJ sequences, and detects clonal expansions by standardized parallel analysis of several aliquots (20). The applicable regulations for volunteer stem cell donors precluded the acquisition of additional epidemiological information such as dwelling and other living conditions that are associated with allergy. The observed frequency of 0.09% IgE-expressing cells among peripheral B cells is on the high end of the reported spectrum (23, 24). The consequences of a possible overestimation of the true prevalence of IgE+ B cells would predominantly imply that the sequencing of the IgE repertoires would have been even more exhaustive than indicated by our simulations. In this context, presence of clonally related IgE sequences in several cellular aliquots is a much more reliable indicator of clonal expansions than BCR sequence read counts in massive parallel sequencing experiments. Nevertheless, it is a striking observation that IgE sequences found in all 5 aliquots dominated the sequence libraries (not shown), suggesting that few IgE+ clones actually had expanded strongly.

The presence of SHM indicates that IgE^+^ B cells have passed through germinal center reactions. The intermediate SHM load of IgE between IgM and IgG/IgA could result from direct CSR of the majority of healthy donor PBMC from IgM to IgE (13). Alternatively, non-IgE B cells could have acquired higher SHM loads by repeated GC passages, whereas IgE^+^ B cells have only a limited presence in germinal centers, (25–27). The difference in IgE mutation rate compared to other isotypes was more striking than in another recent study of IgE in non-allergic subjects, yet not as low as in children with atopic dermatitis (15, 16). These findings call for further verification.

The striking lack of clonal relationships between IgE-expressing B cells and B cells expressing the other isotypes supports the important conclusion that IgE-expressing B cells emerge from qualitatively different immune responses. While the IgE repertoire has been sequenced to apparent exhaustion in our study, incomplete sampling of the other isotype compartments cannot completely exclude a low degree of clonal overlap. However, lack of clonal overlap is corroborated by a recent massive parallel sequencing study that also found virtually no clonal relationship between IgE and other isotypes in healthy donors (16). In contrast, marked overlap of allergen-specific IgE clones with IgG and IgA has been described in allergic individuals (14, 17, 28) and in immunized mice (13).

The distinct characteristics of the normal IgE repertoire, i.e., relatively low SHM burden, lack of intraclonal sequence variation, skewed usage of IGHV genes, marked oligoclonality, retention of germline-encoded NLGM, frequent acquisition of additional such motifs, and striking absence of clonal relatedness to IgM-expressing and non-IgE-class-switched B cells cumulatively indicates its origin from qualitatively different immune responses than IgG- and IgA-expressing B cells. Like allergic individuals (15, 17), healthy donors appear to have IgE repertoires composed of a small pool of highly expanded clones. However, in healthy donors, these likely represent rearrangements in a low affinity, non-antigen-specific, “static” state. As previously suggested, such low-affinity IgE could have a protective role against allergy by competition with high-affinity, type I hypersensitivity-inducing IgE for occupation of Fcε receptors (13, 29).

The novel hypothesis of a specific role of N-glycosylation in expansion and maintenance of IgE-expressing B cells in non-allergic individuals warrants further studies. Ubiquitous NLGM acquisition could have a disease-specific role in selection of IgE-expressing B-cells in similarity to follicular lymphoma (30), primary cutaneous follicle center lymphoma (31) and rheumatoid arthritis (32). On the other hand, glycosylation at NLGM positions could non-specifically obstruct antigen recognition and effectively inhibit BCR affinity maturation (33).

Our findings also support direct class switch recombination from IgM to IgE as the origin of such low-affinity IgE antibodies as demonstrated in mouse studies (10, 13, 19).

Some, but not all (17, 18, 28), previous studies found preferential IGHV usage in allergic individuals, most notably of IGHV2, IGHV4, and IGHV5 family genes (15, 34–36). Our data do not indicate this particular IGHV bias in healthy individuals. Although theoretically attributable to study population differences, primer binding bias in multiplexed primer strategies creates an inherent risk of skewing repertoire analyses. Use of a forward primer binding to an artificial uniform sequence at the 5′ cDNA termini effectively alleviates this risk (7, 20, 31, 37). Differential amplification efficiency of different IGHV genes may be another theoretical source of bias for observed IGHV usage. However, IgE sequences underwent only two additional thermocycles than the other isotypes, effectively limiting this particular risk. In addition, ARTISAN PCR employs long extension times to avoid such bias. In previous applications of this method, no preferential V allele amplification was actually observed (20).

In comparison to allergic individuals and derivatives of high-affinity murine clones, healthy donors appear to carry IgE sequences with fewer BCR mutations (12–15, 18, 28) and less restriction to few individual clonotypes (7, 12, 38, 39). Although IgE VDJ from allergic and parasite-exposed patients also lacked evidence of substantial antigenic selection (7, 15), these characteristics indicate that high-affinity (allergen-specific) IgE clones in allergic individuals are probably generated through indirect class switch recombination from IgG and IgA clonal expansions, preferentially utilizing a restricted repertoire of IGHV genes (13).

Overall, our results add various new aspects to the current knowledge on the IgE repertoire (7). Future projects investigating IgE repertoires in allergic disease should be generated by the same high-standard unbiased approach used here to allow for side-to-side comparison with healthy donors.

## Ethics Statement

This study was carried out in accordance with the guidelines as outlined by the LUMC Biobank Committee with written informed consent from all subjects. All subjects gave written informed consent in accordance with the Declaration of Helsinki. The protocol was approved by the LUMC Medical Ethical Testing Committee.

## Author Contributions

CvB obtained samples. MK and IT obtained the data. MK analyzed the data. DA, MN, and SK provided bioinformatics support. MK and HV wrote the manuscript. All authors read and approved the manuscript.

### Conflict of Interest Statement

The authors declare that the research was conducted in the absence of any commercial or financial relationships that could be construed as a potential conflict of interest.
