# Supplementary material for: Peripheral IgE Repertoires of Healthy Donors Carry Moderate Mutation Loads and Do Not Overlap With Other Isotypes
Source: Front Immunol. 2019 Jul 3;10:1543. doi: 10.3389/fimmu.2019.01543 (PMC6617986; doi:10.3389/fimmu.2019.01543)
Supplement: Supplementary file 2 [file Data_Sheet_2.docx]

**Supplement to:**

**Peripheral IgE Repertoires of Healthy Donors Carry Moderate Mutation Loads and Do Not Overlap With Other Isotypes**

Marvyn Koning^1^, Ignis J.M. Trollmann^1^, Cornelis A.M. van Bergen^1^, Diego Alvarez Saravia^2^, Marcelo A. Navarrete^2^, Szymon M. Kiełbasa^3^, Hendrik Veelken^1^

Departments of ^1^Hematology and ^3^Biomedical Data Sciences, Leiden University Medical Center, Leiden, The Netherlands

^2^School of Medicine, University of Magallanes, Punta Arenas, Chile

**Correspondence:**

Prof. Dr. H. Veelken

Leiden University Medical Center

Albinusdreef 2

2333ZA Leiden

The Netherlands.

Telephone: +31 71 526 2267

Fax: +31 71 526 6755

E-mail: [j.h.veelken@lumc.nl](mailto:j.h.veelken@lumc.nl)

**The authors have no conflicts of interest to disclose.**

**Supplementary methods and data: An *in silico* model to assess false positive identification of clonally related expansions**

In contrast to our results, an earlier study that reported 15% overlap between IgE and IgG/IgA clonal expansions.^1^ However, that study permitted up to 20% differences in CDR3 sequence to identify clonal expansions. Conversely, our much more restricted definition readily identified numerous overlapping clonal expansions, but only across non-IgE isotypes. To model the effects of relaxation of clonality criteria, we simulated a random BCR repertoire *in silico* that reflects structural characteristics observed in full-length sequenced BCR. When IgE dataset of Looney *et al.* was simulated and subsequently analyzed by their own parameters, 12% of sequences were false-positively assigned to clonal expansions. In contrast, analysis of the same dataset with our parameters reduced false positives to zero. Therefore, we conclude that application of relaxed clonality criteria leads to severe overestimation of clonal relationships across BCR isotypes and confirms the absence of clonal relationships between IgE and non-IgE repertoires. This conclusion is corroborated by a previous study investigating IgE repertoires in bone marrow.^2^

**Generation of *in silico* repertoires**

In silico repertoires were generated to compare the frequency of falsely identified clonal relationships between unrelated BCR rearrangements. From all productive IgM, IgG and IgA VDJ sequences obtained from the 6 healthy donors in this study, the following results were analysed from IMGT/HighV-QUEST output data:

1. V gene usage
2. Number of germline nucleotides removed from the 3’ end of the V gene
3. Number of N nucleotides added to the 3’ end of the V gene
4. D gene usage
5. Number of germline nucleotides removed from the 5’ end of the D gene
6. Number of N nucleotides added to the 5’ end of the D gene
7. Number of germline nucleotides removed from the 3’ end of the D gene
8. Number of N nucleotides added to the 3’ end of the D gene
9. J gene usage
10. Number of germline nucleotides removed from the 5’ end of the J gene
11. Number of N nucleotides added to the 5’ end of the J gene

A script was written to create de novo, in silico rearranged BCR by the following functions:

Randomly call V sequences according to the proportions found in result [1]. Remove a number of characters from the right end of the V string. The number of characters to be removed is randomly obtained from result [2] according to the frequencies in which each length was found. To the right end of the V string, add random occurrences of “A”, “C”, “T” and “G” . The number of characters to be removed is randomly obtained from result [3] according to the frequencies in which each length was found.

In a separate file, randomly call D sequences according to the proportions found in result [4]. Remove a number of characters from the left end of the D string. The number of characters to be removed is randomly obtained from result [5] according to the frequencies in which each length was found. Remove a number of characters from the right end of the D string. The number of characters to be removed is randomly obtained from result [7] according to the frequencies in which each length was found. When the sum of [5] and [7] is equal or greater than the length of the string, the entire D string is to be replaced by a 0-character string. If not: to the left end of the D string, add random occurrences of “A”, “C”, “T” and “G” . The number of characters to be removed is randomly obtained from result [6] according to the frequencies in which each length was found. To the right end of the D string, add random occurrences of “A”, “C”, “T” and “G” . The number of characters to be removed is randomly obtained from result [8] according to the frequencies in which each length was found.

3In a separate file, randomly call J sequences according to the proportions found in result [9]. Remove a number of characters from the left end of the J string. The number of characters to be removed is randomly obtained from result [10] according to the frequencies in which each length was found. To the left end of the J string, add random occurrences of “A”, “C”, “T” and “G” . The number of characters to be removed is randomly obtained from result [11] according to the frequencies in which each length was found.

Concatenate V ,D and J strings.

Ergo: ([1] –[2] +[3]) + ([4] – [5] – [7] + [6] + [8]) + ([9] - [10] + [11]).

The resulting rearrangements were then analysed by IMGT/HighV-QUEST for productivity. On average, 20,97% of rearrangements were found to be productive, which is comparable to the 20% estimated by Rajewski in his landmark 1996 paper.^3^

To recreate the results obtained by Looney *et al*, 163 productive *in silico* rearrangements were created and called ‘IgE’ whilst another 255,333 rearrangements were created and called ‘non-IgE’. We then used Geneious v10.1.3 software to identify clonal relationships between each of the rearrangements in the ‘IgE’ dataset and any of the sequences in the ‘non-IgE’ dataset. For the method of clonality calling described by Looney et al, we looked for sequences with identical V genes and a CDR3 nucleotide difference of ≤10,2% (the 20% described in their paper minus twice the median degree of somatic hypermutation: 4,9%). For our own method, we identified sequences with identical V, D and J genes, identical CDR3 length and and a CDR3 nucleotide difference of ≤5%. With the Looney et al method, 19/164 sequences from the ‘IgE’ dataset were false positively correlated with the ‘non-IgE’ dataset, corresponding to 12% of sequences. With our parameters, no sequences were false-positively attributed to a clonal expansion.

**Supplementary Figures**

**Supplementary Figure 1**

Shown is the percentage of unique identifiers sampled per amount of oversampling, i.e. if 5000 sequences were obtained from a sample of 1000 B cells, this amounts to 5x oversampling. Black vertical bars indicate the range of values obtained from 100 simulations.


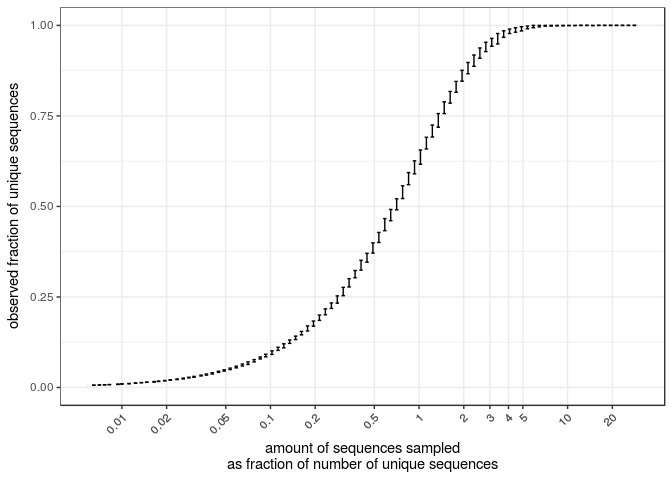


**Supplementary references**

1. Looney TJ, Lee JY, Roskin KM, et al. Human B-cell isotype switching origins of IgE. *J Allergy Clin Immunol.* 2016;137(2):579-586 e577.

2. Levin M, Levander F, Palmason R, Greiff L, Ohlin M. Antibody-encoding repertoires of bone marrow and peripheral blood-a focus on IgE. *J Allergy Clin Immunol.* 2017;139(3):1026-1030.

3. Rajewsky K. Clonal selection and learning in the antibody system. *Nature.* 1996;381(6585):751-758.
